# Computational Design of an mRNA Vaccine Targeting LRP6 for Triple‐Negative Breast Cancer Therapy

**DOI:** 10.1002/cnr2.70567

**Published:** 2026-05-05

**Authors:** Pooriya Teimoori, Mohammadreza Heidari

**Affiliations:** ^1^ Department of Biotechnology, School of Pharmacy Alborz University of Medical Sciences Karaj Iran; ^2^ Department of Clinical Pharmacy, School of Pharmacy Alborz University of Medical Sciences Karaj Iran

**Keywords:** immunotherapy, LRP6, mRNA vaccine, triple‐negative breast cancer

## Abstract

**Background:**

Triple‐negative breast cancer (TNBC) presents a poorer prognosis than other breast cancer subtypes, attributed to its aggressive nature and the lack of specific therapeutic interventions. TNBC has high recurrence rates and limited survival despite current therapies, emphasizing the critical need for improved treatment options. TNBC exhibits increased levels of LRP6 expression, which is linked to tumor‐related features such as growth, metastasis, poor prognosis, resistance to chemotherapy, and invasion. Therefore, LRP6 offers a promising option for therapeutic intervention in breast cancer.

**Aims:**

This research aims to use in silico and bioinformatics techniques to develop an mRNA vaccine that specifically targets the LRP6 antigen.

**Methods and Results:**

The final vaccine construct comprised 431 amino acids, with a molecular weight of 47.5 kDa, theoretical pI of 5.11, and an instability index of 38.3 indicating stability. Population coverage analysis showed broad global coverage of 99.04%. Molecular docking revealed strong binding affinities to immune receptors, including HLA‐A0201 (−812.0), HLA‐A0301 (−707.1), HLA‐DRB1*0101 (−955.7), and TLR9 (−1339.5). Immune simulation predicted high titers of IgG1 antibodies, sustained memory B cell populations (> 200 by Day 365), elevated CD4+ T cells (> 3000), and robust IFN‐γ responses. Codon optimization yielded a high CAI value of 0.94 and GC content of 58.37%, supporting efficient expression in human systems.

**Conclusion:**

Collectively, these results suggest that the designed LRP6‐targeted mRNA vaccine could induce durable humoral and cellular immunity against TNBC and warrants further experimental validation.

## 1 Introduction

Globally, breast cancer stands out as a leading cause of female mortality, accounting for 24.5% of all carcinomas and having a mortality‐to‐incidence ratio of 15.5%. In 2020, it surpassed lung cancer as the most commonly diagnosed kind of cancer. Triple‐negative breast Cancer (TNBC) is a distinct subtype of breast cancer that is identified by the absence of estrogen, progesterone, and HER2 receptors. It constitutes around 10%–15% of all occurrences of breast cancer [1]. Currently, chemotherapy and radiotherapy are the primary treatments, but they have limited efficacy and several side effects [2]. Therefore, it is crucial to the development of efficient therapies.

Low‐density lipoprotein receptor‐related protein 6 (LRP6), a transmembrane receptor, is a vital element in the Wnt signaling pathway, playing a crucial role in embryonic development, maintaining tissue balance, and promoting cell proliferation [3]. Activation of the Wnt/β‐catenin pathway occurs when a Wnt ligand interacts with the Frizzled receptor and its co‐receptor, LRP6 [4]. The disruption of the Wnt signaling pathway has been linked to the onset and advancement of various cancer types, such as pancreatic cancer, breast cancer, colorectal cancer, and hepatocellular carcinoma. Targeted therapies aimed at Wnt/β‐catenin signaling are under investigation, demonstrating potential in both preclinical and clinical studies for the treatment of malignant tumors [5]. These tumors exhibit increased levels of LRP6 expression, which is linked to tumor‐related features such as growth, metastasis, poor prognosis, resistance to chemotherapy, and invasion [6, 7]. The expression of LRP6 is increased in both human TNBC patients and cell lines [8]. Thus, LRP6 has the potential to serve as a specific molecular target for breast cancer treatment.

Preclinical and clinical studies indicate that LRP6 is overexpressed in TNBC and can promote tumor migration, invasion, and metastatic phenotypes via enhanced Wnt/β‐catenin signaling activity [8]. Bioinformatic analyses reveal elevated LRP6 mRNA in tumors with correlations to poor overall survival and disease‐free intervals in several cancers, alongside associations with functional states such as angiogenesis and drug sensitivity. Therapeutic strategies include pharmacological inhibitors (e.g., Niclosamide), natural compounds (e.g., curcumin), non‐coding RNAs, proteins, and peptides that downregulate LRP6 or disrupt its interactions, highlighting advantages over broader Wnt inhibitors, such as reduced side effects. This positions LRP6 as a multifaceted target for overcoming resistance and advancing personalized cancer treatments [9]. While LRP6 is ubiquitously expressed (including in normal tissues as a Wnt co‐receptor), targeted inhibition shows manageable side effects in preclinical models, though vaccine approaches require careful epitope selection to minimize autoimmunity risks.

Cancer vaccines are a form of immunotherapy that activates the immune system to identify and eradicate cancer cells. FDA‐approved examples encompass the human papillomavirus (HPV) vaccine, which averts cervical cancer and other malignancies resulting from HPV infection, and the Sipuleucel‐T vaccination, employed for the management of metastatic prostate cancer [10, 11]. Further, cancer vaccines have shown promise in clinical trials for various cancers but are not yet widely used in clinical practice. Research is focused on developing new and more effective cancer vaccines, combining them with other immunotherapies like checkpoint inhibitors [10].

mRNA vaccines are a promising approach for preventing infectious diseases and cancer. In contrast to conventional vaccines, which entail weakened or inactivated pathogens or their components, mRNA vaccines utilize a fragment of genetic material known as messenger RNA (mRNA) to direct cells in synthesizing a protein that stimulates an immune reaction. This method presents numerous benefits, such as accelerated development and manufacturing, enhanced safety, and increased adaptability in targeting pathogens and cancer antigens [12].

In this study, we employed an integrated immunoinformatics pipeline to design a multi‐epitope mRNA vaccine targeting LRP6. We systematically predicted and screened epitopes for cytotoxic T lymphocytes (CTL), helper T lymphocytes (HTL), and linear B lymphocytes (LBL) based on antigenicity, immunogenicity, toxicity, and allergenicity. These epitopes were assembled into a single vaccine construct using established linker and adjuvant sequences. The construct was evaluated for antigenic potential, safety features, structural stability, and molecular interactions with immune receptors, and in silico immune simulations were performed to predict its ability to elicit effective antitumor immune responses.

## 2 Material and Methods

### 2.1 LRP6 Expression in TNBC Patient Cohorts

#### 2.1.1 TCGA Data Acquisition and Processing

Transcriptomic and clinical data for triple‐negative breast cancer patients were retrieved from The Cancer Genome Atlas (TCGA‐BRCA) cohort through the UCSC Xena Browser (https://xenabrowser.net; accessed on January 26, 2026) [13]. TNBC samples were defined as tumors lacking estrogen receptor (ER), progesterone receptor (PR), and HER2 expression based on TCGA clinical annotations. Normalized RNA‐sequencing data (normalized count + 1) were used to assess LRP6 gene expression. Patients were further categorized according to the basal‐like molecular subtype, as annotated in the TCGA dataset. Comparisons of LRP6 expression were performed between TNBC and non‐TNBC breast cancer samples, and between basal‐like and non–basal‐like TNBC subgroups. Statistical differences were evaluated using Welch's *t*‐test implemented in the UCSC Xena platform.

#### 2.1.2 Immune Correlation Analysis

To investigate the relationship between LRP6 and immune‐related markers, correlation analysis was conducted using TCGA‐BRCA RNA‐sequencing data. Normalized gene expression values (norm_count + 1) were employed. A panel of immune‐associated genes was selected, including TLR2, TLR4, TLR7, TLR9, CD8A, CD8B, GZMB, PRF1, CD247, HLA‐A, HLA‐DRA, and HLA‐DRB1. Pairwise correlations between LRP6 and these immune marker genes were calculated using Pearson correlation analysis.

### 2.2 Retrieval of the Target Protein Sequence and Analysis

Data on amino acid sequence, extracellular domain, and cellular localization were obtained from the UniProt server (https://www.uniprot.org/; accessed on June 8, 2025) [14] (UniProt ID: O75581). The protein antigenicity evaluation was conducted using the VaxiJen server (http://www.ddg‐pharmfac.net/vaxijen/VaxiJen/VaxiJen.html; accessed on June 8, 2025) [15, 16, 17]. VaxiJen employs an alignment‐free methodology grounded in auto‐covariance (ACC) transformation to achieve precise antigen prediction (70%–89%). It categorizes antigens based solely on their physicochemical properties, preceding sequence alignment, thereby enhancing efficiency in predicting protective antigens [15]. TMHMM server—2.0 (https://services.healthtech.dtu.dk/services/TMHMM‐2.0/; accessed on June 8, 2025) [18, 19] was utilized to evaluate protein transmembrane topology. The Alphafold server (https://alphafold.ebi.ac.uk/; accessed on June 8, 2025) [20] was employed to predict the protein's 3D structure.

### 2.3 Prediction and Identification of Optimal Immunogenic Epitopes

#### 2.3.1 CTL Epitope Prediction

The Immune Epitope Database (IEDB) server (https://www.iedb.org/; accessed on June 8, 2025) [21] was employed for predicting MHC I epitopes, utilizing an extensive array of algorithms including Stabilized Matrix Method (SMM) [22], SMM with Peptide and Artificial Neural Network (ANN) [23]: MHC Binding Energy Covariance matrix (SMMPMBEC), Scoring Matrices derived from Combinatorial Peptide Libraries [24], Consensus [25], NetMHCpan [26], NetMHCcons [27], PickPocket [28], and NetMHCstabpan [29]. The prediction was conducted with a percentile rank below 2. Different HLAs, such as HLA‐A01:01, HLA‐A02:01, HLA‐A02:06, HLA‐A03:01, HLA‐A24:02, HLA‐A26:01, HLA‐B07:02, HLA‐B08:01, HLA‐B27:05, HLA‐B39:01, HLA‐B44:02, HLA‐B44:03, HLA‐B58:01, HLA‐B58:02, were considered for epitope predictions, with a standard epitope length of nine amino acids. IEDB is a robust resource for epitope prediction, boasting comprehensive experimental data across diverse immune contexts. Integrating external resources like UniProt and NCBI Taxonomy enhances accuracy. The IEDB server employs quantitative algorithms for MHC class I epitope prediction, rigorously evaluated through blind prediction tests for accuracy and reliability in vaccine development and immunotherapy [21, 30].

Epitopes underwent antigenicity assessment using VaxiJen2.0, employing a threshold > 1. Allergenicity was evaluated via AllerCatPro 2.0 (https://allercatpro.bii.a‐star.edu.sg/; accessed on June 8, 2025) [31] and AllerTOP v. 2.0 (https://www.ddg‐pharmfac.net/AllerTOP/; accessed on June 8, 2025) [32], complemented by toxicity prediction through the Toxinpred server (https://webs.iiitd.edu.in/raghava/toxinpred/algo.php; accessed on June 8, 2025) [33, 34] utilizing the SVM method (SVM (Swiss‐Prot) + Motif based) with a threshold set at −0.5. The IEDB immunogenicity prediction tool (http://tools.iedb.org/immunogenicity/; accessed on June 8, 2025) [35] was utilized for epitope immunogenicity prediction, with recommended masking positions at the default (first, second, and C‐terminus amino acids) and a threshold set at a score greater than 0.2. The AllerCatPro 2.0 server utilizes algorithms incorporating amino acid sequences and 3D structures to predict protein allergenicity. It attains a high accuracy of 84.7% [31]. The AllerTOP v. 2 server employs machine learning algorithms such as multilayer perceptron (MLP), naïve Bayes (NB), logistic regression, decision tree, random forest, and k nearest neighbors (kNN) to predict allergens with high accuracy. It achieves an overall accuracy of 88.7% using these algorithms [32]. The ToxinPred server utilizes advanced algorithms to predict peptide toxicity accurately. With accuracy rates reaching 90% and above, the server's algorithms demonstrate exceptional precision in distinguishing toxic and non‐toxic peptides [33]. The IEDB Immunogenicity server utilizes algorithms to forecast the immunogenicity of peptides on HLA class I molecules. The model has shown notable accuracy, with approximately 66% of immunogenic pMHCs receiving a positive score, compared to 44% for non‐immunogenic pMHCs on average [35].

#### 2.3.2 HTL Epitope Prediction

The IEDB server [21], utilizing algorithms like Consensus [36], Combinatorial library, NN‐align‐2.3 (netMHCII‐2.3) [37], SMM‐align (netMHCII‐1.1) [38], and Sturniolo [39], predicted MHC II epitopes with a Percentile Rank > 2. NetMHCIIpan‐4.1 BA (https://services.healthtech.dtu.dk/services/NetMHCIIpan‐4.0/; accessed on June 8, 2025) [40] was also employed, with thresholds set for strong binders (% Rank) at 1 and weak binders (% Rank) at 5. The length of the predicted epitopes was 15 amino acids, and epitopes were predicted on all HLAs of the NetMHCIIpan‐4.1 BA server. The NetMHCIIpan‐4.0 server uses the NNAlign_MA and NNAlign_MAC models, significantly improving accuracy in predicting CD4+ neoepitopes. The accuracy is approximately 70%–80% [40].

The predicted epitopes' antigenicity, allergenicity, and toxicity were evaluated using VaxiJen2.0, AllerCatPro 2.0, AllerTop v. 2.0, and ToxinPred servers, respectively, with the same criteria for CTLs. Furthermore, the IFNepitope (https://webs.iiitd.edu.in/raghava/ifnepitope/application.php; accessed on June 8, 2025) [41] and IL4pred (http://crdd.osdd.net/raghava/il4pred/; accessed on June 8, 2025) [42] servers were employed to screen epitopes for their ability to induce Interferon‐gamma and Interleukin‐4, respectively. The IFNepitope server employs a combined motif and SVM strategy to forecast IFN‐gamma epitopes, featuring distinct models tailored for IFN‐gamma versus non‐IFN‐gamma and IFN‐gamma versus other cytokines. Meanwhile, the IL4pred server adopts a hybrid (SVM + Motif) approach to predict IL4‐inducing epitopes, with an SVM threshold established at 0.2. The IFNepitope server employs machine learning methodologies, encompassing Support Vector Machine (SVM) models utilizing peptide amino acid and dipeptide composition. Through this hybrid methodology, the server achieved a peak prediction accuracy of 82.10% [41]. The IL4pred server employs motif‐based and machine learning algorithms, incorporating support vector machine (SVM) models alongside a hybrid approach. These prediction models demonstrate an accuracy rate of 78.76% [42].

#### 2.3.3 Docking of Epitopes With HLAs

Epitope docking with HLAs in vaccine design ensures precise antigen presentation prediction, identifying optimal epitopes and enhancing immune response efficacy by maximizing immunogenicity and HLA compatibility [43]. Epitope docking was performed using the HDOCK server (http://hdock.phys.hust.edu.cn/; accessed on June 8, 2025) [44, 45, 46, 47, 48, 49], which entailed matching HTL epitopes with HLA DRB1 0101 (PDB ID: 1AQD) and CTL epitopes with HLA A 0201 (PDB ID: 3MRG). Five HTL and CTL epitopes with the highest docking scores were chosen for further analysis.

#### 2.3.4 Linear B Cell Epitope Prediction

The IEDB server was utilized for the prediction of linear B‐cell epitopes, employing methods such as Bepipred Linear Epitope Prediction 2.0 [50, 51, 52], Kolaskar and Tongaonkar Antigenicity [53] methods (threshold: 1), Bepipred Linear Epitope Prediction [54] and Emini Surface Accessibility Prediction [55] (threshold: 0.5). Furthermore, the ABCpred server (https://webs.iiitd.edu.in/raghava/abcpred/index.html; accessed on June 8, 2025) [56, 57], with a threshold of 0.51, was employed to forecast linear B‐cell epitopes with a length of 16 amino acids. The ABCpred server utilizes a recurrent neural network (Jordan network) for epitope prediction and demonstrates an approximate accuracy of 65.93% in predicting B cell epitopes. Like HTLs and CTLs, predicted epitopes underwent comprehensive screening for antigenicity, allergenicity, and toxicity using VaxiJen2.0 (threshold > 0.5), AllerCatPro 2.0, AllerTop v. 2.0, and ToxinPred servers. Additionally, the IgPred server (https://webs.iiitd.edu.in/raghava/igpred/; accessed on June 8, 2025) was utilized to assess epitopes and identify those capable of inducing immunoglobulins G (IgG), A (IgA), and E (IgE) [58]. The final selection comprised five epitopes.

### 2.4 Evaluation of Population Coverage

Assessing the population coverage of T cell epitopes in vaccine design is essential for understanding genetic diversity and the prevalence of HLA alleles. The IEDB Population Coverage server (http://tools.iedb.org/population/; accessed on June 8, 2025) is pivotal in this analysis [59].

### 2.5 Construction of the Vaccine Structure

The mRNA vaccine construct consists of multiple parts arranged from 5′ to 3′, encompassing the 5′ Cap, 5′UTR of human Beta Globin (NCBI ID: NM 000518.5), Kozak sequence, a signal peptide of tissue Plasminogen Activator (tPA) (UniProt ID: P00750), Pan HLA‐DR Binding Epitope (PADRE), GM‐CSF as an adjuvant, CTL, HTL, and LBL epitopes, MHC class I tracking domain (MITD) (UniProt ID: Q8WV92), stop codon, 3′UTR, and poly‐A tail (120 A). Diverse models were constructed employing distinct linkers (EAAAK, AAY, GPGPG, GSG, GSSG, and KK) and subjected to Protparam server (https://web.expasy.org/protparam/; accessed on June 8, 2025) [60]. The ProtParam server computes various protein properties, including amino acid and atomic composition, molecular weight, extinction coefficient, theoretical isoelectric point (pI), half‐life, aliphatic index, instability index, and GRAVY. The instability index gauges the protein's stability in a laboratory setting, with a value below 40 indicating stability and above 40 indicating potential instability [60]. All the analyses were performed on the translatable part of the vaccine structure, which ultimately makes up its main protein structure (from PADRE to the end of LBL epitopes).

### 2.6 Computational Modeling of the 2D and 3D Structure of the mRNA Vaccine

The Robetta server (https://robetta.bakerlab.org/submit.php; accessed on June 8, 2025) predicted the 3D structure of all three designed vaccine structures, Utilizing automated domain detection and modeling protocols, including homology modeling and de novo methods [61]. For each structure, three of the best‐predicted models were selected and refined with GalaxyRifine (https://galaxy.seoklab.org/cgi‐bin/submit.cgi?type=REFINE; accessed on June 8, 2025) [62, 63]. Among the nine refined models, four of the best models were selected and re‐refined with both GalaxyRifine and Modrefiner servers (https://zhanggroup.org/ModRefiner/; accessed on June 8, 2025) [64]. Finally, the best model among these four was selected.

The models were chosen based on assessments conducted by the SAVES server (https://saves.mbi.ucla.edu/; accessed on June 8, 2025), including ProCheck [65], ERRAT [66], and Verify3D [67, 68] tools. ERRAT uses pairwise interactions to identify errors in protein structures [66]. Verify3D evaluates protein structure quality by examining the compatibility between a 3D model and its corresponding amino acid sequence [68]. The ProCheck method uses stereochemical considerations to assess the overall quality of a protein structure and identify regions that may require further investigation [65]. After acquiring the ultimate model, additional analyses were conducted to assess antigenicity, allergenicity, and toxicity. These evaluations utilized VaxiJen2.0, AllerCatPro 2.0, AllerTop v. 2.0, and ToxinPred servers.

### 2.7 Analysis of Solubility and Other Physicochemical Properties

As previously stated, the Protparam server was employed to evaluate the physicochemical properties of the vaccine structure. Furthermore, solubility was assessed using the Protein‐Sol server (https://protein‐sol.manchester.ac.uk/; accessed on June 8, 2025) [69]. The Protein‐Sol algorithm computes 35 sequence features, encompassing amino acid composition, sequence length, and composite scores such as KmR and DmE. Additional features cover folding and disorder propensities, beta‐strand propensities, hydropathy, pI, sequence entropy, and absolute charge at pH 7 [69].

### 2.8 Evaluation of Post‐Translational Protein Nature

NetPhos 3.1 (https://services.healthtech.dtu.dk/services/NetPhos‐3.1/; accessed on June 8, 2025) [70], NetNGlyc—1.0 (https://services.healthtech.dtu.dk/services/NetNGlyc‐1.0/; accessed on June 8, 2025) [71], MyrPS/NMT server (https://mendel.imp.ac.at/myristate/; accessed on June 8, 2025) [72], the Big‐PI/GPI Animals server (https://mendel.imp.ac.at/gpi/gpi_server.html; accessed on June 8, 2025) [73, 74, 75], and PrePS server (https://mendel.imp.ac.at/PrePS/; accessed on June 8, 2025) [76] are employed to predict phosphorylation, N‐glycosylation, myristoylation, glycosylphosphatidylinositol (GPI) modification, and prenylation, respectively.

### 2.9 2D Protein Structure Prediction

Utilizing the PSIPRED server (http://bioinf.cs.ucl.ac.uk/psipred/; accessed on June 8, 2025) [77], the 2D protein structural model of the chosen model was forecasted using a two‐stage neural network algorithm reliant on position‐specific scoring matrices produced by PSI‐BLAST. The PSIPRED server attained an average Q3 score of 76.5%–78.3% for precise secondary structure prediction [77].

### 2.10 2D mRNA Structure Prediction

The predicted secondary structure of the mRNA vaccine was generated utilizing the RNAfold web server, part of the ViennaRNA Package 2.0 (http://rna.tbi.univie.ac.at/cgi‐bin/RNAWebSuite/RNAfold.cgi; accessed on June 8, 2025). RNAfold predicts the minimum free energy secondary structure of single RNA sequences using dynamic programming, and additionally computes equilibrium base‐pairing probabilities via partition function algorithms, providing insights into RNA structural ensembles and thermodynamic stability [78].

### 2.11 Conformational B Cell Epitopes in Vaccine Structure

The Ellipro tool (http://tools.iedb.org/ellipro/; accessed on June 8, 2025) [79] from the IEDB server was utilized to forecast discontinuous B cell epitopes of the formulated vaccine. The default settings for the Minimum Score and Maximum Distance parameters were applied. The Ellipro server utilizes three algorithms: one that approximates protein shape as an ellipsoid, another that calculates the residue protrusion index, and a third that clusters neighboring residues based on their protrusion index values. Collectively, these algorithms achieve an accuracy rate of 0.832 [79].

### 2.12 Computational Models for Immune System Simulation

The C‐IMMSIM server (https://kraken.iac.rm.cnr.it/C‐IMMSIM/; accessed on June 8, 2025) [80] was utilized for in silico modeling of the immune system's reaction after administering the developed vaccine. This model integrates a three‐dimensional stochastic cellular automaton and incorporates bioinformatics tools to forecast interactions among immune system components. It employs methods such as NetMHCPan and Miyazawa‐Jernigan potential, with an average accuracy of 56% correlation with experimental data [80]. A random seed of 12 345, a simulation volume of 10, a total of 1095 simulation steps, and a specific host HLA selection (A0101 and B0702 for MHC class I and DRB1: 0101 for MHC class II) were all used in conjunction with the C‐IMMSIM server. In addition, the vaccination was administered in three doses, separated by 28 days between shots.

### 2.13 Unraveling Molecular Docking of the Vaccine Peptide

The constructed vaccine underwent docking simulations with seven molecules, namely HLA‐A02 (PDB: 3MRG), HLA‐A03 (PDB: 2XPG), HLA‐DRB1 0101 (PDB: 1AQD), TLR2 (PDB: 2Z7X), TLR4 (PDB: G8A), TLR7 (PDB: 7CYN), and TLR9 (PDB: Q9NR96), utilizing the ClusPro server for in‐depth molecular interaction analysis. The ClusPro server (https://cluspro.bu.edu/login.php?redir=/queue.php; accessed on June 8, 2025) employs advanced algorithms to achieve PIPER, FFT, and Monte Carlo to attain high‐quality outcomes. It provides the PIPER energies of cluster centers alongside the minimum PIPER energy within each cluster, aiding in the anticipation of protein complexes [81, 82, 83, 84]. The PDBsum server (https://www.ebi.ac.uk/thornton‐srv/databases/pdbsum/; accessed on June 8, 2025) was utilized to evaluate the interactions within the docked structures of the vaccine with MHC I, MHC II, TLR2, TLR4, TLR7, and TLR9. PDBsum provides comprehensive structural annotations of docked protein complexes by integrating data from PDB entries, employing algorithms like HBPLUS and LIGPLOT to visualize interactions, offering insights into interface residues, hydrogen bonds, and ligand contacts [85].

### 2.14 Host‐Specific Codon Optimization of the Vaccine Construct

To enhance translational efficiency in human cells, codon optimization was performed on the peptide vaccine construct using the GenSmart Codon Optimization tool provided by GenScript (https://www.genscript.com/tools/gensmartcodon‐optimization; accessed on June 8, 2025). The coding sequence corresponding to the region expressed in the host, spanning from the signal peptide to the MITD segment, was optimized specifically for *Homo sapiens* as the target expression system. The optimized DNA sequence was further analyzed using the GenSmart Rare Codon Analysis platform provided by GenScript (https://www.genscript.com/tools/rare‐codon‐analysis; accessed on June 8, 2025) to assess its suitability for expression in human cells. The analysis provided key parameters related to expression efficiency in human cells, including Codon Adaptation Index (CAI), Codon Frequency Distribution (CFD), and GC content. CAI reflects the overall compatibility of the codon usage with the host system, while CFD indicates the presence of rare codon clusters that may affect translational efficiency. GC content was also assessed to ensure structural stability and suitability for transcriptional processing.

### 2.15 Statistical Analysis

Welch's *t*‐test was used for TCGA gene expression comparisons via the UCSC Xena platform, and Pearson correlation analysis was applied to assess associations between LRP6 expression and immune‐related marker genes, including Toll‐like receptors (TLRs), CD8‐associated genes, cytotoxic markers, and HLA genes. For vaccine epitope selection, Python (v3.12.3) was used to analyze antigenicity, toxicity, allergenicity, immunogenicity, and other predictive features. Epitopes were systematically filtered according to predefined thresholds described in the relevant Methods sections to ensure objective, transparent, and reproducible selection. Descriptive statistics were used to summarize epitope scores, docking energies, and immune simulation outputs. Data processing, correlation analysis, visualization, and descriptive analyses were conducted using Python. Graphical outputs generated by external platforms (e.g., UCSC Xena, IEDB tools, and immune simulation servers) were used as provided by the respective tools. Machine learning algorithms embedded within external bioinformatics servers are described in their corresponding methodological subsections.

## 3 Results

### 3.1 LRP6 Expression in TNBC Patient Cohorts

#### 3.1.1 LRP6 Expression in TNBC and Basal‐Like Subtypes

**FIGURE 1:**
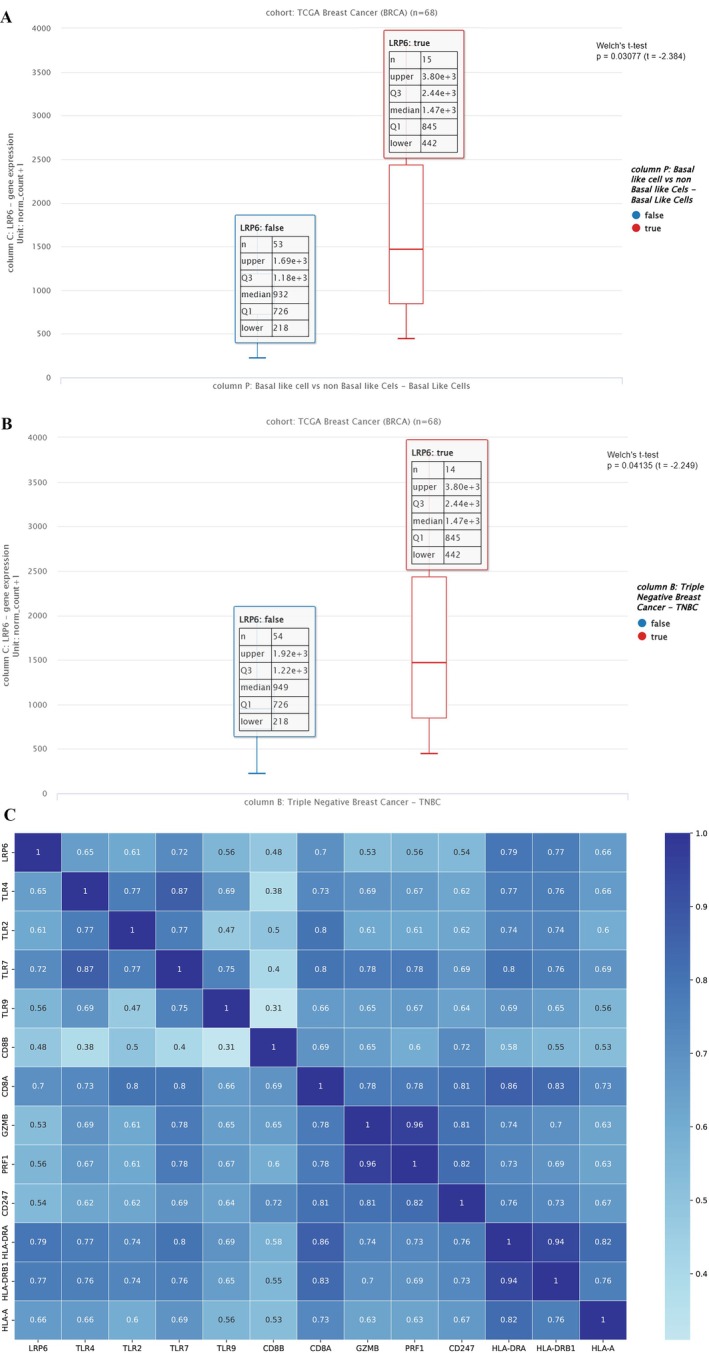
(A) LRP6 expression and immune correlations in TCGA‐BRCA. (B) LRP6 expression in TNBC versus non‐TNBC tumors. (C) Heatmap of Pearson correlations between LRP6 and immune‐related genes.

Analysis of TCGA‐BRCA RNA‐sequencing data revealed that LRP6 expression was significantly higher in triple‐negative breast cancer (TNBC) tumors compared with non‐TNBC breast cancers (Welch's *t*‐test, *p* = 0.041). TNBC samples displayed a higher median LRP6 expression and a wider distribution, suggesting greater heterogeneity within this subtype. Similarly, basal‐like tumors exhibited significantly elevated LRP6 expression compared with non–basal‐like tumors (*p* = 0.030) (Figure 1).

#### 3.1.2 Immune Correlation Analysis

Correlation analysis between LRP6 and immune‐related genes was visualized in Figure 1. LRP6 expression showed strong positive correlations with several immune markers, including TLR7, CD8A, HLA‐DRA, and HLA‐DRB1. These results suggest that elevated LRP6 expression is associated with enhanced expression of immune‐related genes in TNBC.

### 3.2 Target Protein Analysis

**FIGURE 2:**
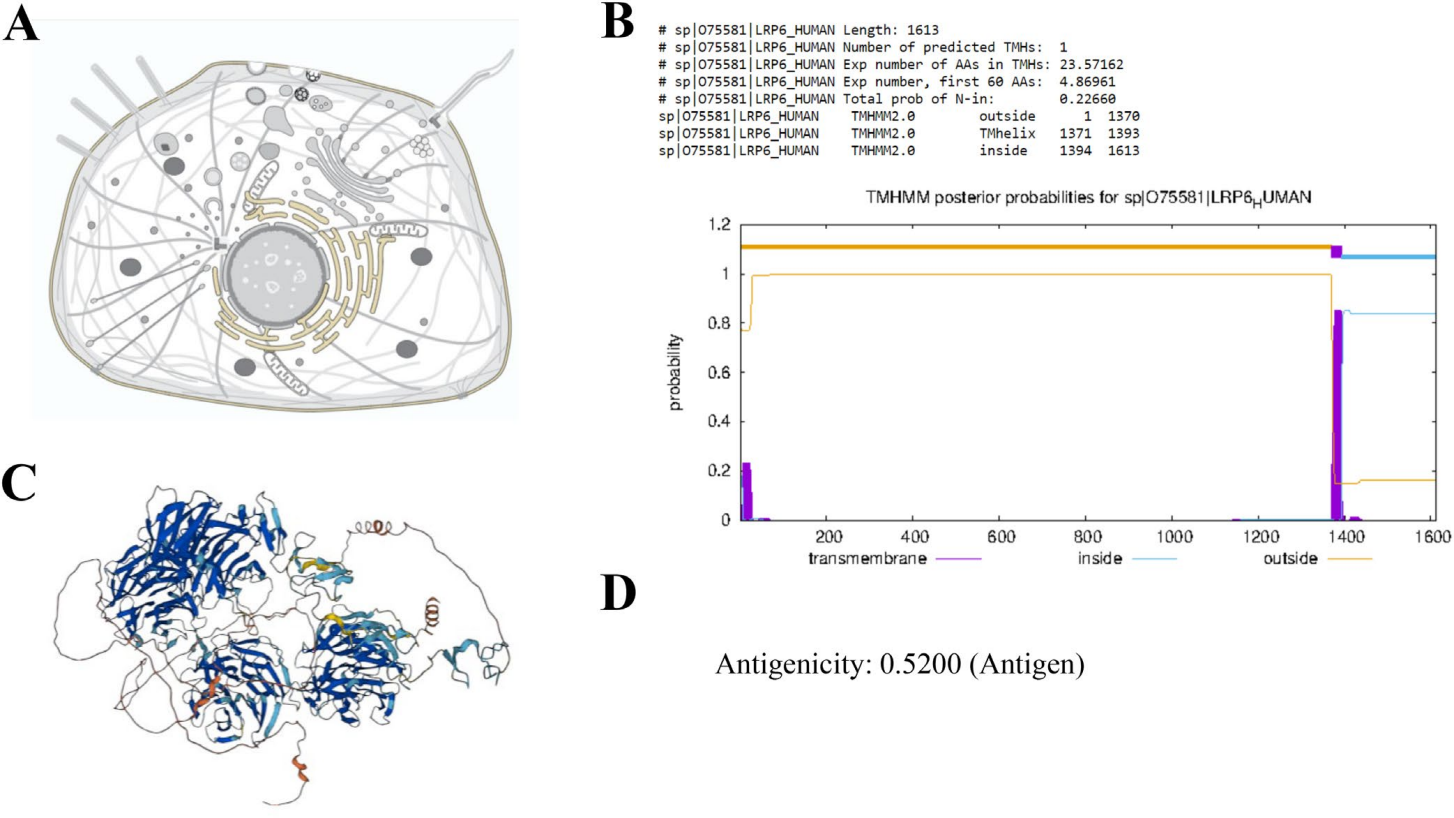
Target protein analysis: (A) The cellular placement of LRP6 was acquired from the Uniprot database. (B) The protein's transmembrane arrangement was obtained through analysis from the TMHMM server—2.0. (C) The three‐dimensional structure of LRP6 was acquired using the Alphafold server. (D) The evaluation of antigenicity for LRP6 was obtained using the Vaxijen 2.0 tool.

Data regarding the LRP6 target protein was procured from the UniProt server. Examination conducted using VaxiJen2.0 and TMHMM server—2.0 revealed that LRP6 is classified as a membrane protein and exhibits acceptable antigenicity. Figure 2 elucidates the outcomes obtained from the servers and the three‐dimensional structure of LRP6.

### 3.3 Assessment and Examination of the Most Optimal Epitopes

#### 3.3.1 Assessment of CTL Epitopes

The IEDB server utilized various algorithms to successfully predict 450 non‐repetitive CTL epitopes with a length of 9aa. According to the findings from the VaxiJen2.0 server, it was observed that there were 88 epitopes with antigenicity greater than one. Among these 88 epitopes, 75 were confirmed by the Toxinpred server regarding toxicity. Furthermore, the IEDB server demonstrated that out of the 77 epitopes, 15 possessed the essential characteristics of immunogenicity. Five epitopes were chosen after analyzing the docking outcomes through the HDOCK server and considering the allergenicity assessment conducted by the AllerCatPro 2.0 and AllerTop v 2.0 servers. These epitopes demonstrated non‐allergenic properties and exhibited the lowest docking scores (Table 1).

**TABLE 1:** Analysis of CTL, HTL, and LBL epitopes of LRP6.

Epitopes	Number	Sequence	Antigenicity	Toxicity	Allergenicity	Other features	
CTL						Docking score	Immunogenicity
	1	DKIPHIFGF	1.4412	−0.98	Non‐allergen	−216.42	0.30742
	2	SRYIYWTCE	1.1326	−0.56	Non‐allergen	−200.44	0.33889
	3	RRADIRRIS	1.2196	−1.06	Non‐allergen	−195.72	0.33478
	4	RYIYWTCEA	1.2547	−0.76	Non‐allergen	−194.51	0.30472
	5	DEVRAIRRS	1.8925	−0.7	Non‐allergen	−176.22	0.30278

HTL						Docking score	INF Gama	IL4
	1	PHPFGLTQYQDYIYW	1.1907	−1.39	Non‐allergen	−265.91	0.70115593	0.21
	2	PHPFALTLFEDILYW	1.2095	−0.86	Non‐allergen	−232.11	0.20108865	0.25
	3	DFTDIVLQLEDIRHA	1.3371	−0.97	Non‐allergen	−236.59	0.11713919	0.3
	4	EGRTKVQARIAQLSD	1.3389	−1.51	Non‐allergen	−218.1	0.15279	0.55
	5	RYIYWTCEATNVINV	1.2018	−0.67	Non‐allergen	−216.8	0.21367255	0.28

LBL						Ig prediction
	1	IADDLPHPFGLTQYQDYIYW	1.0800	−1.2	Non‐allergen	IgG epitope
	2	WIDKQQQMIEKIDMTGREGRTKVQ	0.9526	−0.68	Non‐allergen	IgA epitope
	3	DWGEIPKIERAALDGS	0.7052	−1.27	Non‐allergen	IgG epitope
	4	DLSIDIYSRYIYWTCE	0.6371	−1.1	Non‐allergen	IgG epitope
	5	DYVYWTDWQRRSIERVHKRSAEREV	0.5946	−1.64	Non‐allergen	IgG epitope

#### 3.3.2 Investigation of HTL Epitopes

Interrogations on the IEDB server utilizing various algorithms and the NetMHCIIpan‐4.1 BA server unveiled 971 Helper T Lymphocyte (HTL) epitopes, each comprising 15 amino acids. Assessment of these epitopes via the VaxiJen2.0 server indicated that out of the 971 epitopes identified, 110 demonstrated antigenicity scores surpassing 1. Analysis of epitope toxicity using the ToxinPred server indicated that 96 of the identified epitopes exhibited non‐toxic characteristics. Subsequent analysis utilizing the IL4pred and IFNepitope servers revealed that 25 epitopes among the identified 96 demonstrated the potential to induce both IL‐4 and IFN‐gamma responses. Ultimately, five epitopes were chosen for further evaluation following the evaluation of docking results using the HDOCK server and the allergenicity assessment conducted by the AllerCatPro 2.0 and AllerTop v 2.0 servers (Table 1).

#### 3.3.3 Analysis of LBL Epitopes

Utilizing two servers, IEDB with diverse algorithms and the ABCpred server, predictions for B‐cell linear epitopes were conducted, yielding a total of 238 identified epitopes. All of them were analyzed using VaxiJen2.0, revealing that 149 exhibited antigenicity above 0.5 H. Of these 149 epitopes, 121 were approved for toxicity by the Toxinpred server. Following this, the IgPred server was utilized to evaluate the epitopes for the induction of different antibody classes, affirming 11 instances. Subsequently, five epitopes demonstrating the highest antigenicity were chosen (Table 1).

### 3.4 Population Coverage Analysis

**FIGURE 3:**
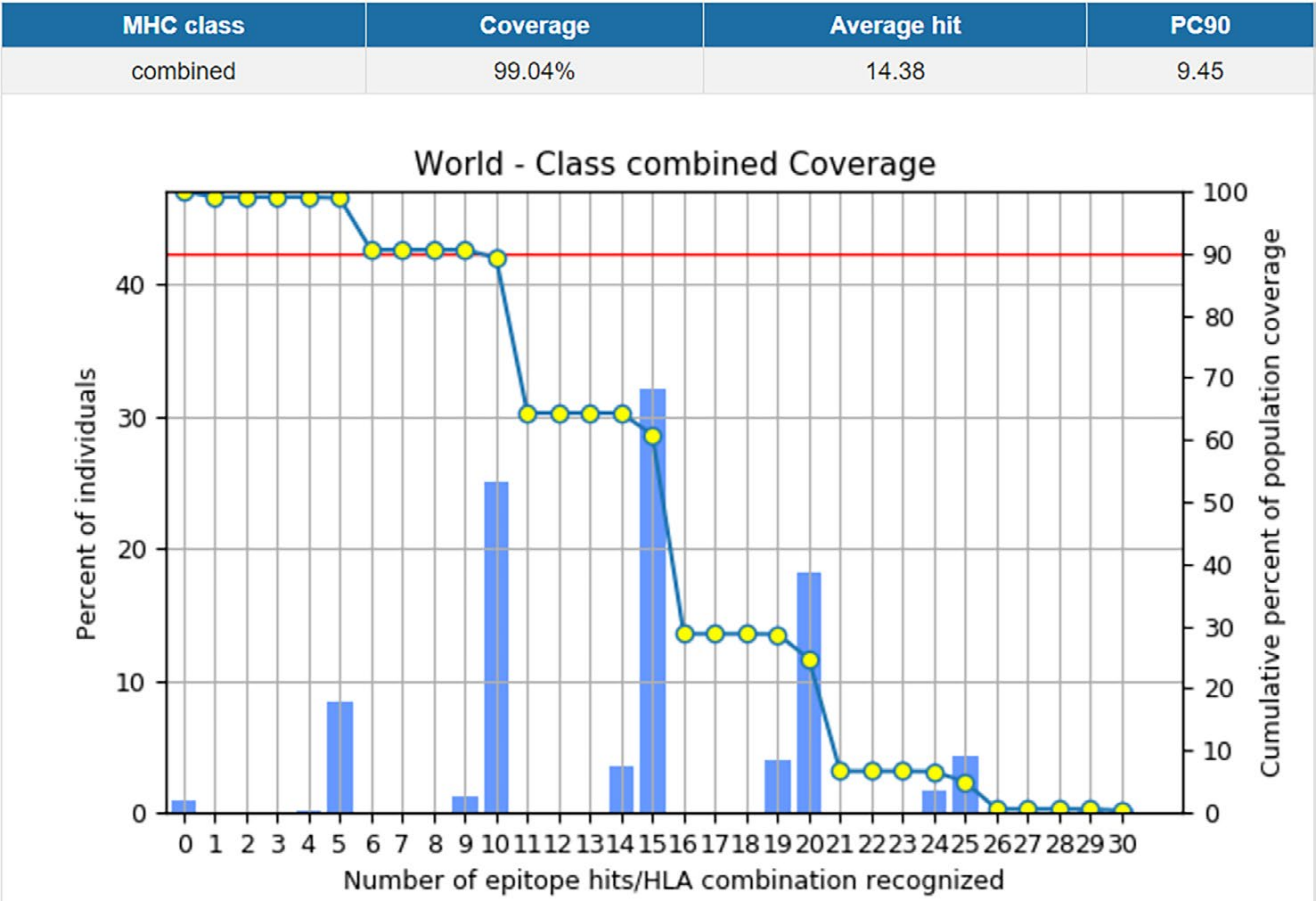
Combined Population Coverage of MHC I and II result.

Based on the findings presented in Figure 3, the analysis of the population coverage of MHC I and II epitopes conducted by the IEDB server indicated a comprehensive combined coverage of 99.04%.

### 3.5 Designing mRNA Vaccine and Analysis

**FIGURE 4:**
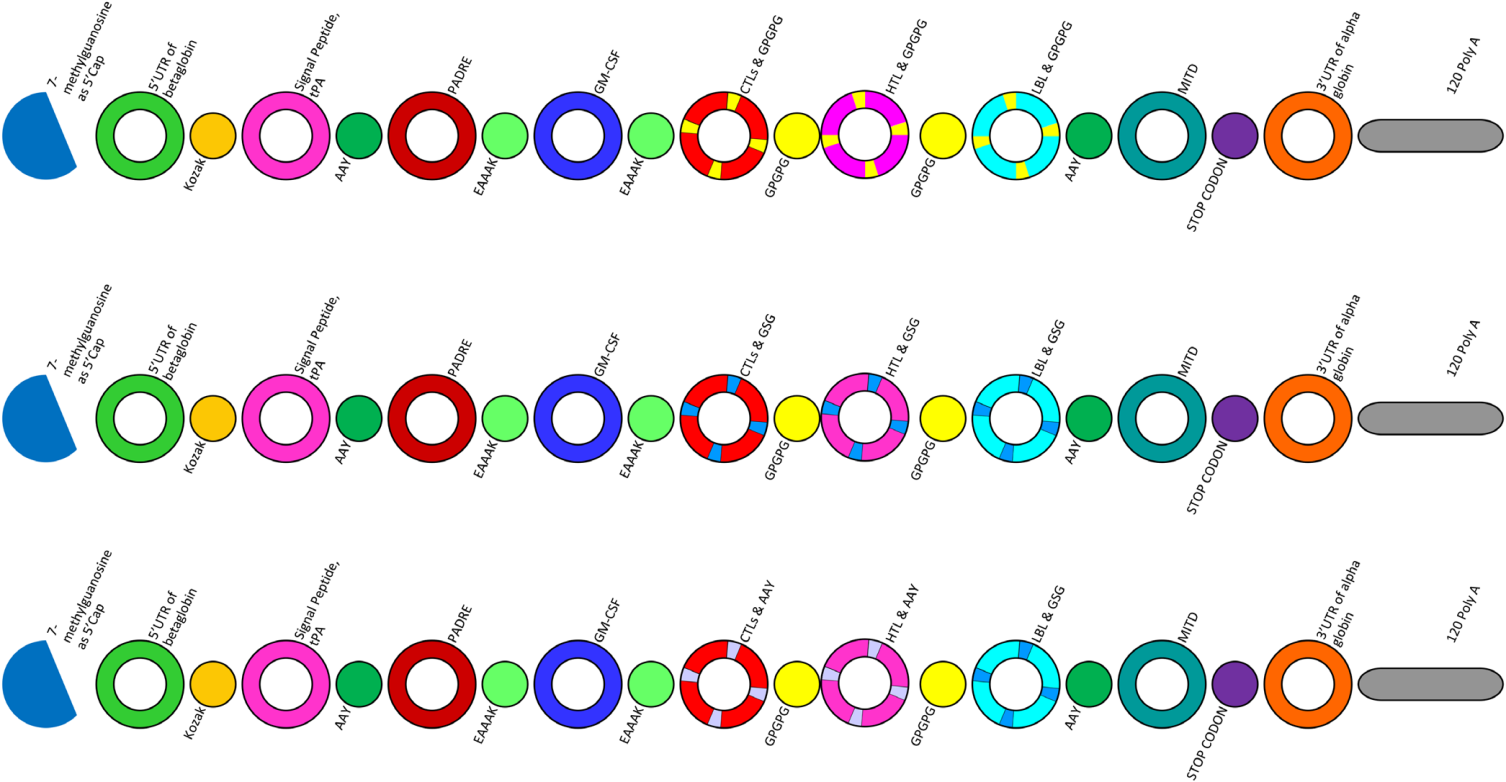
Three vaccine models designed with different linkers that had an Instability Index below 40.

The vaccine components were joined using various linkers and assessed using the Protparam server. Three models with an Instability Index lower than 40 were chosen for further progress (Figure 4, Table 2).

**TABLE 2:** Three models of designed vaccines with their linkers and instability indexes.

Model	Likers	Instability index
1	EAAK, GPGPG	38.30
2	EAAK, GPGPG, GSG	39.96
3	EAAK, GPGPG, GSG, AAY	39.67

### 3.6 3D Modeling and Refinement

**FIGURE 5:**
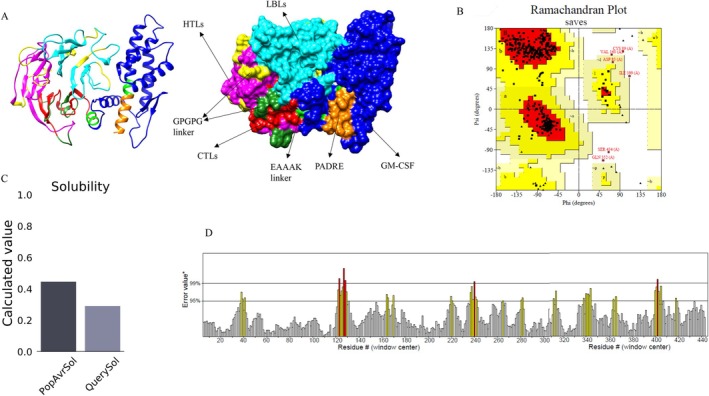
Overview of Constructed vaccine analysis: (A) 3D structure of constructed vaccine. (B) Ramachandran Plot of the vaccine provided by ProCheck tool. (C) Solubility chart of protein‐sol server. (D) Result of ERRAT tool which represents bad contacts in vaccine structure.

Three designed models were constructed and subjected to 3D modeling using the Robetta server, generating five structures for each model. After careful evaluation based on the assessment of ProCheck results for each predicted structure, specific structures (1, 2, and 4) from the first model, (1, 2, and 3) from the second model, and (1, 3, and 5) from the third model were chosen. Subsequently, these selected structures underwent refinement using the GalaxyRifine server. ProCheck analysis identified four optimal items, which were further re‐refined by both GalaxyRefine and Modrefiner servers. A comparison of results using the ProCheck server revealed superior outcomes from the GalaxyRifine server. Ultimately, among the four structures refined by the GalaxyRefine server, the first structure from the first model exhibited the most favorable results and was chosen for subsequent stages. The software Chimera 1.171.1 was employed to depict the three‐dimensional structure of the vaccine visually (Figure 5A).

### 3.7 Protein Structure Validation

The findings of the Ramachandran plot from the ProCheck tool revealed that most residues, accounting for 91.0%, were present in the most favored regions. Additionally, a proportion of 7.3% were situated within additional allowed regions, while a smaller percentage of 1.8% were located in other regions (Figure 5B). The overall quality factor was determined to be 90.55 (Figure 5D). A total of 78.17% of the residues demonstrate an average 3D‐1D score equal to or greater than 0.1. Assessments from AllerCatPro 2.0, AllerTop v. 2.0, and ToxinPred confirm the non‐allergenic and non‐toxic nature of the constructed vaccine. Additionally, the VaxiJenserver calculated an antigenicity value of 0.6596.

The in silico validation results of the constructed vaccine's 3D model are provided in the Table S1.

### 3.8 Protein Characteristics and Modifications Analysis

As previously indicated, the vaccine models were chosen based on the outcomes obtained from the Protparam server. The outcomes of this server's analysis regarding the most favorable model are summarized in Table 3. The vaccine consists of 431 amino acids and has a molecular weight of 47561.78 Da. The calculated theoretical isoelectric point (pI) of the substance is 5.11. Notably, the anticipated half‐life varies among different organisms: 30 h in mammalian reticulocytes (in vitro), over 20 h in yeast (in vivo), and more than 10 h in *Escherichia coli* (in vivo). The protein's stability is classified based on the computed instability index (II) 38.30. In addition, the vaccine has an aliphatic index of 69.28. The hydropathicity of the substance, as measured by the grand average of hydropathicity (GRAVY), is found to be −0.463, demonstrating its hydrophilic nature. These metrics offer crucial information about the vaccine's structural and biochemical properties, which are essential for its design and potential effectiveness.

**TABLE 3:** Physicochemical properties and post‐translational modifications of constructed vaccine.

Characteristic		Amount		
Chemical composition	Total number of atoms	6594		
	Formula	C_2147_H_3236_N_570_O_624_S_17_		
	Number of amino acids	431		
	Molecular weight	47561.78		
	Theoretical pI	5.11		
	Number of negatively charged residues (Asp + Glu)	51		
	Number of positively charged residues (Arg + Lys)	37		
Post transitional properties	Phosphorylation	Total 37 (16T, 15S, 6Y)		
	N‐glycosylation	Position	Potential	N‐Gly result
		62	NLSR	0.7626
		72	NETV	0.7107
	Myristoylation	No myristoylation site accessible for NMT could be predicted		
	Glycosylphosphatidylinositol (GPI) modification site	Nonpotential GPI‐modification site		
	Prenylation	No Prenylation site		
Protein properties	Instability index	38.30		
	Aliphatic index	69.28		
	Grand average of hydropathicity (GRAVY)	−0.463		
	Antigenicity	0.6596		
	Allergenicity	PROBABLE NON‐ALLERGEN		
	Predicted scaled solubility	0.291 (insoluble)		

The analysis conducted by the Protein‐Sol server offers valuable information regarding the solubility attributes of the designed vaccine. The anticipated scaled solubility is 0.291, which falls below the population average (PopAvrSol) of 0.45. Higher values suggest increased solubility compared to average *E. coli* proteins, with lower values indicating reduced solubility [86] (Figure 5C, Table 3).

The post‐translational analysis of the mRNA vaccine revealed the absence of myristoylation and prenylation sites accessible for N‐myristoyltransferase and CaaX farnesyltransferase/Geranylgeranyltransferase, respectively. No potential GPI modification site was found. Two N‐glycosylation sites and 37 phosphorylation sites (16T, 15S, 6Y) were predicted, indicating potential glycosylation modifications and phosphorylation events. The findings are depicted in (Table 3).

### 3.9 Prediction and Analysis of 2D Protein Structure

**FIGURE 6:**
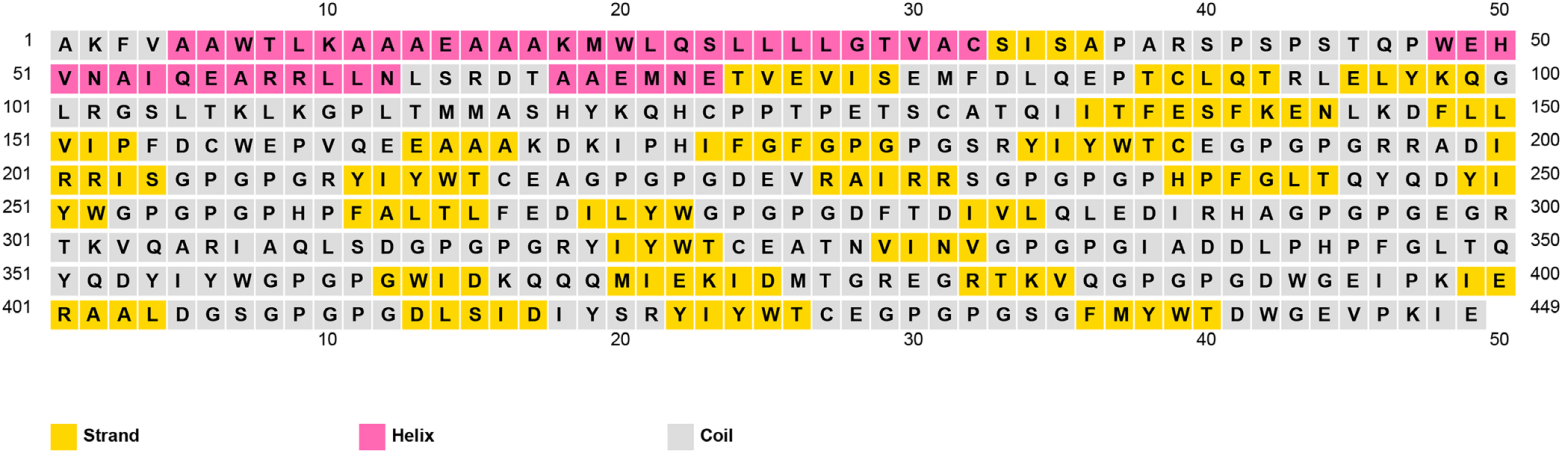
2D structure analysis of the constructed protein vaccine.

The PSIPRED V4.0 algorithm was utilized to forecast the 2D protein structure of the vaccine, as depicted in Figure 6. The structure comprises 10.9% helix and 26.4% strand, with the remaining portion exhibiting coil conformation.

### 3.10 Modeling and Structural Analysis of mRNA Secondary Structure

**FIGURE 7:**
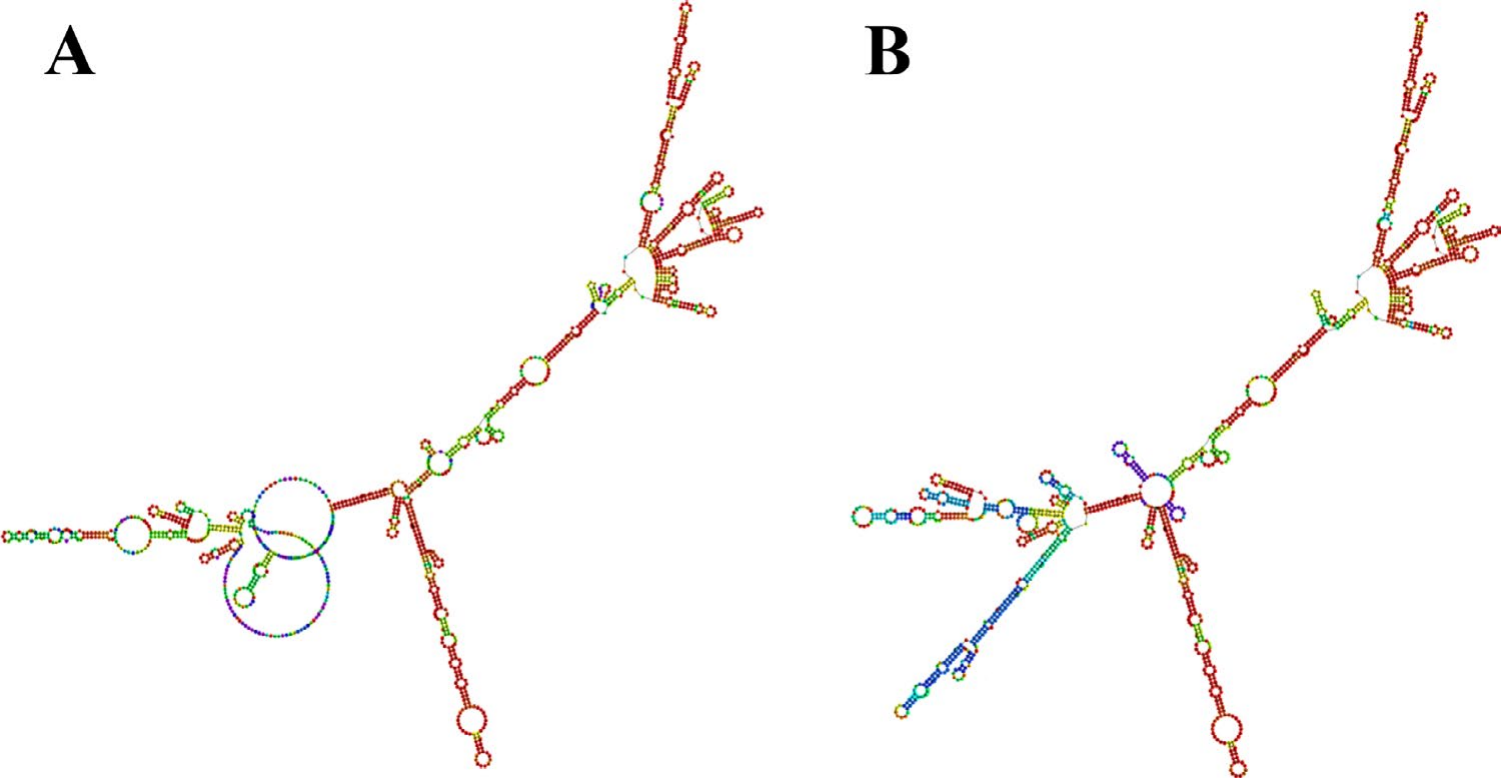
(A) Optimal secondary structure and (B) centroid secondary structure of the mRNA vaccine.

The RNAfold web server was used to predict the secondary structure and assess the thermodynamic stability of the mRNA vaccine. The minimum free energy (MFE) structure showed a stability of −854.30 kcal/mol (Figure 7A), while the centroid structure exhibited −626.84 kcal/mol (Figure 7B). These values reflect a stable and energetically favorable conformation, supporting the structural integrity of the construct.

### 3.11 In Silico Identification of Conformational B‐Cell Epitopes

**FIGURE 8:**
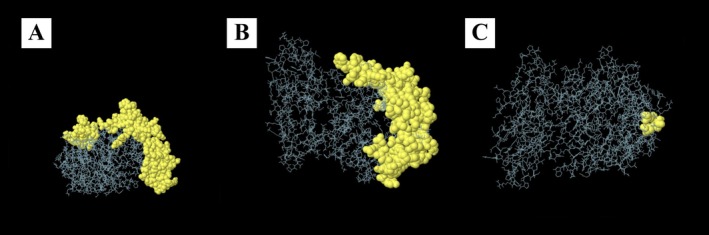
Conformational B cell epitopes of constructed vaccine. (A) First episode (B) second episode (C) third episode.

The Ellipro server anticipated three structural epitopes for B cells on the vaccine structure. The results of this prediction, specifying the locations of these epitopes, are presented in Table 4, and visually depicted in Figure 8.

**TABLE 4:** Conformational B cell epitopes predicted by ellipro server.

No.	Residues	Number of residues	Score
1	A:A1, A:K2, A:F3, A:V4, A:A6, A:G28, A:T29, A:V30, A:A31, A:C32, A:S33, A:I34, A:S35, A:A36, A:P37, A:A38, A:R39, A:S40, A:P41, A:S42, A:P43, A:S44, A:T45, A:Q46, A:P47, A:W48, A:E49, A:H50, A:V51, A:N52, A:A69, A:N72, A:E73, A:V75, A:E76, A:V77, A:I78, A:S79, A:E80, A:M81, A:F82, A:D83, A:L84, A:Q85, A:E86, A:P87, A:T88, A:C89, A:L90, A:Q91, A:T92, A:R93, A:E95, A:L96, A:Y97, A:Q99, A:G100, A:G103, A:S104, A:L105, A:T106, A:K107, A:L108, A:K109, A:G110, A:P111, A:L112, A:T113, A:M114, A:M115, A:A116, A:S117, A:H118, A:K120, A:Q121, A:H122, A:C123, A:P124, A:P125, A:T126, A:P127, A:E128, A:T129, A:S130, A:C131, A:A132, A:T133, A:Q134, A:I135, A:I136, A:T137, A:E139, A:E158, A:P159, A:V160, A:Q161, A:E162, A:G179, A:P180, A:G181, A:S182, A:Y184, A:P206, A:G207, A:P208, A:G209, A:R210, A:Y211, A:R230, A:R231, A:S232, A:G233, A:P234, A:G235, A:P236, A:G237, A:P238, A:H239, A:P240, A:G242, A:L243, A:T244, A:Q245	123	0.747
2	A:D199, A:E217, A:A218, A:G219, A:P220, A:G221, A:P222, A:G223, A:D224, A:E225, A:R227, A:Y246, A:Q247, A:D248, A:Y249, A:I250, A:Y251, A:W252, A:G253, A:P254, A:G255, A:P256, A:G257, A:T264, A:E267, A:I269, A:G275, A:G277, A:D278, A:F279, A:T280, A:D281, A:I282, A:V283, A:L284, A:Q285, A:L286, A:E287, A:H291, A:A292, A:G293, A:P294, A:G295, A:P296, A:G297, A:E298, A:G299, A:R300, A:K302, A:V303, A:Q304, A:A305, A:R306, A:G313, A:P314, A:G315, A:P316, A:G317, A:R318, A:Y319, A:E325, A:A326, A:T327, A:N328, A:V329, A:N331, A:V332, A:G333, A:P334, A:G335, A:P336, A:G337, A:I338, A:A339, A:D340, A:D341, A:L342, A:P343, A:H344, A:Q352, A:D353, A:Y354, A:D365, A:K366, A:Q368, A:M370, A:K373, A:I374, A:D375, A:M376, A:T377, A:G378, A:R379, A:E380, A:G381, A:R382, A:T383, A:K384, A:V385, A:Q386, A:G387, A:P388, A:G389, A:P390, A:G391, A:D392, A:W393, A:G394, A:E395, A:I396	110	0.66
3	A:D288, A:R290, A:T301	3	0.653

### 3.12 Immune Response Analysis Following mRNA Vaccine Injection

**FIGURE 9:**
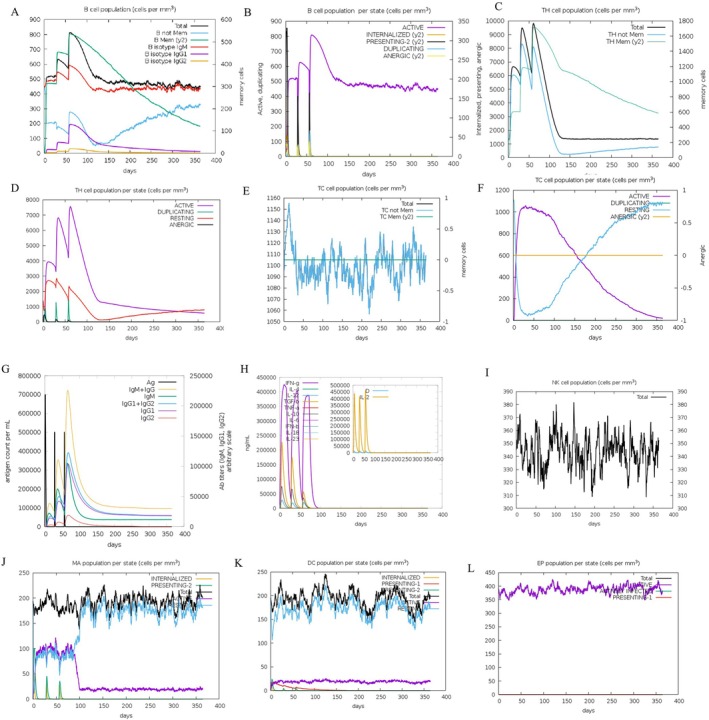
Analysis results of immune system response simulation to vaccine injection. (A) B lymphocytes: Total count, memory cells, and sub‐divided into isotypes IgM, IgG1, and IgG2. (B) B lymphocytes population per entity‐state. (C) CD4 T‐helper lymphocytes count. (D) CD4 T‐helper lymphocytes count sub‐divided per entity‐state. (E) CD8 T‐cytotoxic lymphocytes count. Total and memory shown. (F) CD8 T‐cytotoxic lymphocytes count per entity‐state. (G) The immunoglobulins and the immunocomplexes. (H) Cytokines. Concentration of cytokines and interleukins. (I) Natural Killer cells (total count). (J) Dendritic cells. The total number broken down to active, resting, internalized, and presenting the ag. (K) Macrophages. Total count, internalized, presenting on MHC class II, active, and resting macrophages. (L) Epithelial cells. Total count broken down to active, virus‐infected, and presenting on class I MHC molecule.

The C‐ISSCIM server conducted a comprehensive simulation analysis of the immune system response following three vaccine injections at a 28‐day interval. The assessment of immune system factors extended up to 365 days from the initial injection. The B‐cell populations demonstrated a consistent increase post each injection, sustaining memory B cells above 200 even on Day 365 (Figure 9A). In contrast, highly active B cells maintained at 500 (Figure 9B). Concurrently, T‐cell populations showed an upward trend, with T helper cells surpassing 3000 by Day 365 (Figure 9C) and active T cells exceeding 1000 (Figure 9D–F). These findings suggest the establishment of enduring immunity and vaccine‐induced memory. The IgG concentration surpassed IgM, with IgG1 predominating over IgG2 (Figure 9G). Remarkably, gamma interferon exhibited significant increments post‐injection, followed by IL‐2 and TGF‐β (Figure 9H). The figure also displays data on the macrophage, epithelial cell, DC, and NK cell populations (Figure 9I–L).

### 3.13 Molecular Docking of Vaccine With Immune Targets

**FIGURE 10:**
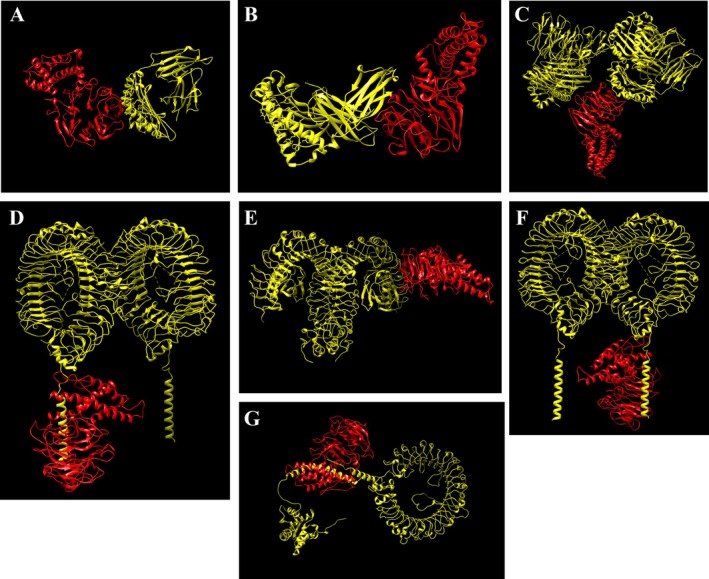
Docking analysis of vaccine with different immune system structures. (A) Docking of HLA A 0201 with vaccine. (B) Docking of HLA A 0301 with vaccine. (C) Docking of HLA DRB1 0101 with vaccine. (D) Docking of TLR 2 with vaccine. (E) Docking of TLR 4 with vaccine. (F) Docking of TLR7 with vaccine. (G) Docking of TLR 9 with vaccine.

The docking outcomes of the vaccine with TLR2, TLR4, TLR7, TLR9, HLA‐A0201, HLA‐A0301, and HLA‐DRB1*0101 molecules, conducted via the ClusPro server, are presented in Table 5. Furthermore, these interactions' three‐dimensional configurations were visualized using Chimera 1.171.1 software, as depicted in Figure 10.

**TABLE 5:** Docking result of ClusPro server.

Receptor	Ligand	Score
TLR 9	Vaccine	−1339.5
TLR 7	Vaccine	−1092.2
TLR 4	Vaccine	−829.5
TLR 2	Vaccine	−821.6
HLA A 0201	Vaccine	−812.0
HLA A 0301	Vaccine	−707.1
HLA DRB1 0101	Vaccine	−955.7

**FIGURE 11:**
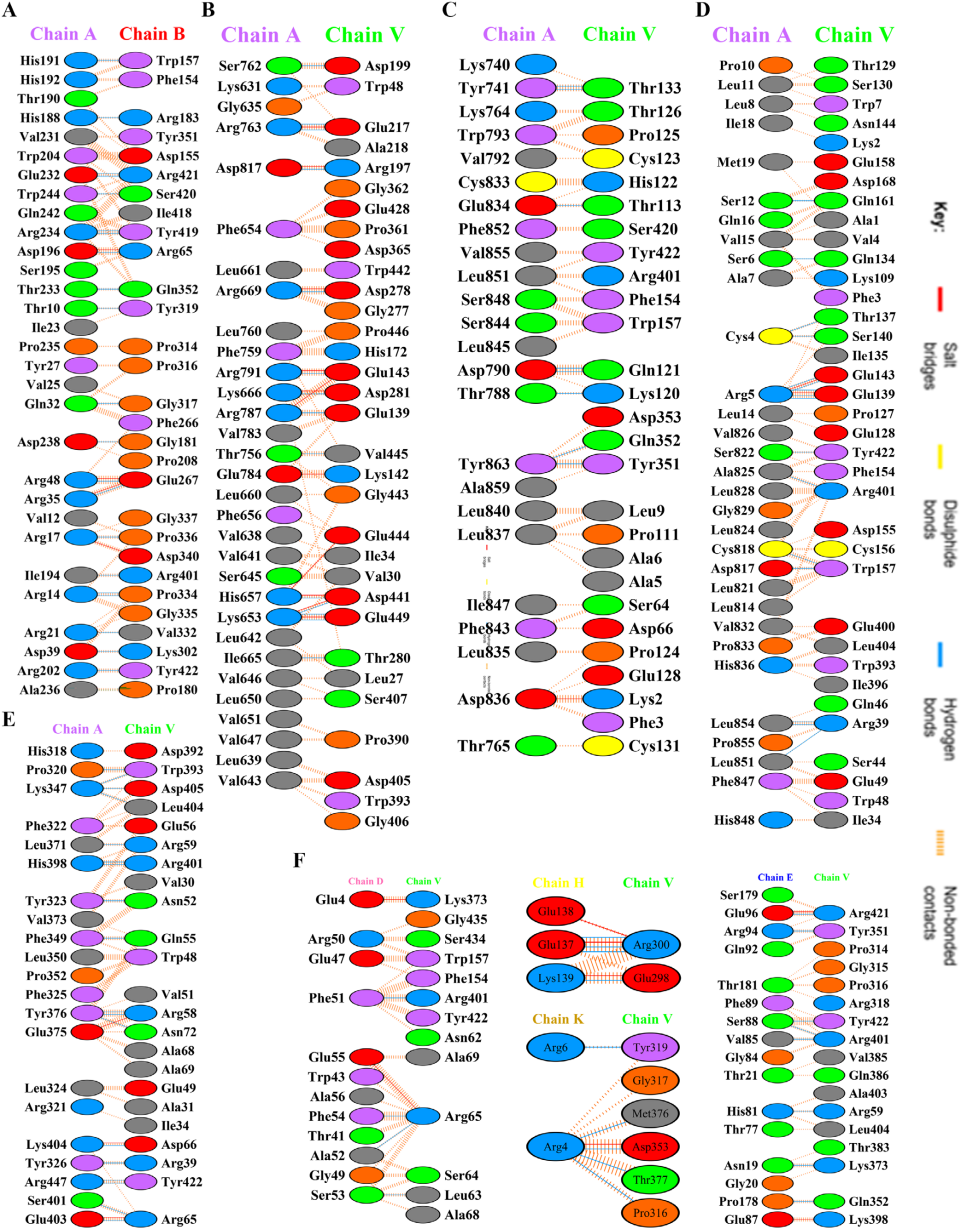
Molecular interactions between chains of docked complexes: (A) MHC I (chain A) and the vaccine construct (chain B); (B) TLR4 (chain A) and the vaccine (chain V); (C) TLR7 (chain A) and the vaccine (chain V); (D) TLR9 (chain A) and the vaccine (chain V); (E) TLR2 (chain A) and the vaccine (chain V); (F) interactions between MHC II chains (D, E, H, K) and the vaccine (chain V).

PDBsum analysis (Figure 11) demonstrates that the designed vaccine establishes strong and diverse interactions with immune receptors. The vaccine–MHC I complex involves 30 and 29 interface residues from MHC I and the vaccine, respectively, with interface areas of 1424 Å^2^ (MHC I) and 1526 Å^2^ (vaccine), stabilized by 4 salt bridges, 21 hydrogen bonds, and 200 non‐bonded contacts. MHC II exhibits chain‐specific variability, with the most extensive interaction observed for Chain E (749 Å^2^ for MHC II, 782 Å^2^ for vaccine), followed by Chain D (475/482 Å^2^), Chain K (192/135 Å^2^), Chain H (118/145 Å^2^), and Chain J (146/146 Å^2^). Among innate immune receptors, TLR4 shows the largest interface area (1889 Å^2^ for TLR4, 1794 Å^2^ for vaccine), supported by 11 salt bridges, 14 hydrogen bonds, and 174 non‐bonded contacts. TLR9 forms a similarly strong interaction (1940 Å^2^ for TLR9, 1777 Å^2^ for vaccine) with 16 hydrogen bonds and 2 salt bridges. TLR7 displays an interface of 1539 Å^2^ (TLR7) and 1406 Å^2^ (vaccine), with 8 hydrogen bonds and 1 salt bridge. TLR2 engages through 1189 Å^2^ (TLR2) and 1183 Å^2^ (vaccine), stabilized by 18 hydrogen bonds, 3 salt bridges, and 157 non‐bonded contacts. These results underline the vaccine's potential to simultaneously activate both adaptive (via MHCs) and innate (via TLRs) immune responses through stable and specific binding.

### 3.14 Codon Optimization and Expression Suitability Analysis

**FIGURE 12:**
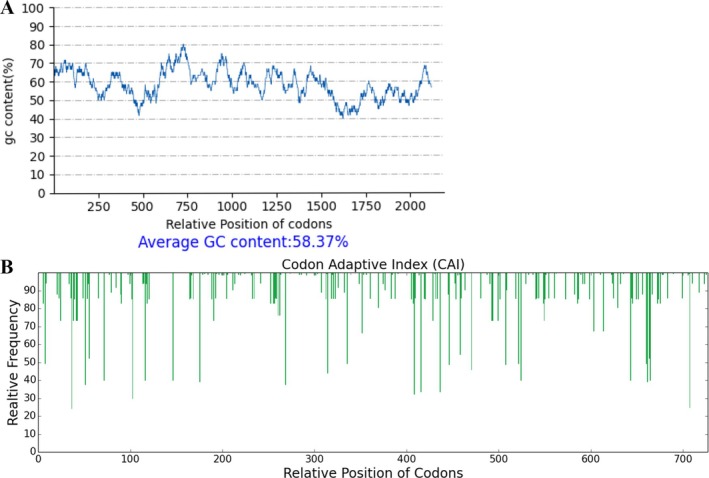
(A) GC content of optimized DNA structure of the vaccine. (B) Codon adaptive index.

Codon optimization of the vaccine DNA sequence was conducted using the GenSmart Codon Optimization tool by GenScript to enhance translational efficiency in human cells. The optimized sequence retained the original length of 2181 base pairs and a GC content of 58.37%, indicating that the optimization process preserved the structural characteristics of the construct (Figure 12A). Subsequently, the optimized sequence was evaluated using the GenSmart Rare Codon Analysis tool. The Codon Adaptation Index (CAI) was calculated to be 0.94 (Figure 12B), suggesting high compatibility with human codon usage, while the Codon Frequency Distribution (CFD) value was 0, indicating the absence of rare codon clusters that could hinder expression.

## 4 Discussion

Among breast cancer subtypes, TNBC is particularly the most aggressive subtype, exhibiting molecular heterogeneity, with distinct subtypes contributing to its aggressiveness and poor prognosis. TNBC has high recurrence rates and limited survival despite current therapies, emphasizing the critical need for improved treatment options [87]. LRP6 high expression has been detected in breast cancer [88]. LRP6 high expression has a positive correlation with both mortality rates and unfavorable prognosis in breast cancer cases. Further Survival analysis revealed a negative correlation between elevated LRP6 expression in breast carcinoma tissue and the overall survival rate [7].

Our computational findings, particularly the elevated expression of LRP6 in TNBC tumors and its association with immune‐related gene signatures, align with existing experimental evidence showing that LRP6 promotes aggressive behaviors in TNBC cells. Previous mechanistic studies demonstrated that LRP6 up‐regulation enhances Wnt/β‐catenin signaling and drives migration and invasion in TNBC cell lines such as MDA‐MB‐231 and BT‐549, while LRP6 inhibition suppresses these malignant phenotypes [8]. Several recent studies have explored vaccine strategies for TNBC, reflecting growing interest in immunotherapeutic approaches despite the historically limited success of conventional treatments in this subtype. A systematic review of cancer vaccines in TNBC highlights early clinical evaluation of RNA‐based neoepitope vaccines, such as RO7198457 combined with the PD‐L1 inhibitor atezolizumab, in patients with advanced solid tumors, including TNBC [89].

Breast cancer vaccines strive to improve the immune system's defense against the disease by employing antigens, delivery mechanisms, and adjuvants to boost immunogenicity. Although various immunogenic proteins have been detected among different types of breast cancer, the majority of antigens consist of weakly immunogenic epitopes, particularly prevalent in triple‐negative breast cancer [90]. Therefore, in silico studies are needed to select the most appropriate antigens. The importance of TNBC vaccine development lies in its potential to revolutionize cancer treatment. Ongoing research in immunoinformatics and computational biology holds promise for advancing this area of cancer immunotherapy [91].

Several recent in silico immunoinformatics studies have pursued multi‐epitope vaccine designs for TNBC, each with distinct antigen choices, design strategies, and predicted immunogenic profiles. A recent multi‐antigen computational study identified several extracellular and intracellular TNBC‐associated proteins, including TROP‐2, EpCAM, MUC‐1, NECTIN4, Mesothelin, MAGE‐A, and NY‐ESO‐1, to design both protein and mRNA vaccine constructs. This design achieved significant predicted population coverage and demonstrated strong in silico immunogenicity, including high epitope binding affinities and immune stimulation potential. The authors proposed a novel four‐part mRNA vaccine regimen to balance epitope diversity with clinical feasibility [92]. Another computational work focused on the MZF‐1 protein as an antigen for metastatic TNBC, finding strong TLR‐4 and TLR‐9 interactions, favorable structural stability, and predicted immune responses, although these designs likewise require in vivo or clinical validation [93]. In contrast to these designs, our study specifically targets LRP6, a Wnt/β‐catenin pathway co‐receptor with oncogenic and immune‐modulatory implications in TNBC. While the other studies predominantly focus on classical tumor‐associated antigens (e.g., MUC‐1, EpCAM, TRIM25), LRP6 represents a mechanistically distinct and less explored immunotherapeutic target. This focus allows us to integrate tumor signaling relevance with immune targeting, which may offer unique therapeutic leverage compared with vaccines targeting general surface markers.

Like several of these studies, we used reverse vaccinology and immunoinformatics tools to predict CTL, HTL, and B‐cell epitopes with high antigenicity and broad HLA coverage. However, unlike designs that prioritize multi‐antigen constructs with potentially large epitope repertoires, our multi‐epitope construct is centered on a single, well‐validated antigen. This targeted strategy may reduce complexity and the risk of off‐target effects while still achieving broad immunogenic potential, particularly because LRP6 overexpression is prevalent in TNBC tumors. Furthermore, population coverage in our design (99.04% for the selected epitopes) is notably high compared with some published studies; for example, the multi‐antigen TNBC design reported 87.75% coverage in the Zahraei et al. study [92]. This suggests that our epitope selection methodology may confer broader applicability across diverse genetic backgrounds.

mRNA vaccines possess notable advantages over recombinant protein vaccines. mRNA vaccines typically have a faster development timeline due to their synthetic nature, enabling rapid adaptation to antigens. They offer the potential for scalability and cost‐effectiveness in manufacturing, as they do not require the intricate protein production processes involved in recombinant protein vaccines. Moreover, mRNA vaccines may elicit robust immune responses, including cellular and humoral immunity. Although mRNA vaccines have challenges, including the need for stringent cold chain storage and distribution and stability, due to their characteristics like safety, efficacy, and versatility, they are a very suitable option for designing cancer vaccines [94].

The process of converting cancer vaccinations into practical treatments that can be widely utilized has posed significant difficulties for many years. Several approaches for cancer vaccination are being developed [95]. The recent authorization of the two mRNA vaccines for COVID‐19 may signal a significant milestone in our pursuit of utilizing mRNA vaccines for cancer immunotherapy [96]. The mRNA technology allows for the precise delivery of antigen‐encoding information directly into host cells, enabling the production of antigenic proteins within the cell's cytoplasm [97]. So, the designed vaccine was an mRNA vaccine. At the beginning of the 5′ segment is 7‐methyl(3‐O‐methyl) GpppG Cap, which protects against exonuclease attacks and promotes translation initiation [98]. The 5′ UTR is vital for ribosome binding, forming the translation initiation complex, and regulating stability [99]. The Kozak sequence, which consists of the consensus sequence ACCATGG, plays a crucial role in translation initiation by eukaryotic ribosomes [100]. Employing tissue plasminogen activator (tPA) as a signal peptide enhances immunogenicity, aiding epitope secretion for robust antigen presentation [101]. PADRE, or pan HLA DR‐binding epitope, is a T‐helper peptide used in vaccine design to induce CD4+ helper T lymphocyte responses [102]. As a cancer vaccine adjuvant, GM‐CSF enhances immune responses by maturing antigen‐presenting cells, stimulating their growth, and influencing CD4+ helper cell subsets [103]. MITD enhances MHC class I epitope presentation, improving CD8+ T cell responses against intracellular pathogens and cancer [104]. The 3′ UTR is crucial for stability, protein expression, and translation efficiency [99]. A long poly(A) tail is crucial in mRNA vaccine design, augmenting translational efficiency and RNA stability. This modification enhances protein expression and cell surface epitope presentation [105].

Antigen detection methods aid in identifying optimal vaccine candidates, facilitating the development of personalized immunotherapy strategies [91]. Predicting MHC I and II epitopes in vaccine design is crucial for identifying specific antigenic targets and stimulating cellular and humoral immune responses. This enhances vaccine specificity, efficacy, and safety, crucial for combating complex diseases. Accurate epitope prediction contributes to developing highly effective vaccines with minimal side effects [106, 107]. Further conformational epitopes provide information on the specific three‐dimensional structures recognized by antibodies, aiding in precisely identifying antigenic regions [108]. Also, the docking technique in vaccine design allows for the prediction of protein–protein interactions, aiding in identifying potential antigen–antibody binding sites, which is crucial for understanding immune responses and designing effective vaccines [83]. In the present study, these in silico and bioinformatics techniques showed promising results for this mRNA candidate targeting LRP6 in TNBC.

Protein translational modifications (PTMs), such as phosphorylation and glycosylation, impact cancer immunogenicity, influencing interactions with the immune system and guiding immunotherapeutic strategies [109]. Prediction of GPI modification and myristoylation in vaccine design can provide advantages such as enhanced immunogenicity, improved antigen presentation, and increased vaccine stability [110]. Prenylation, involving the attachment of isoprenyl lipids to proteins, enhances membrane association, crucial for cell signaling, vesicle trafficking, and cell‐cycle regulation. Targeting prenylation shows promise in cancers and cerebrovascular diseases drug development [111]. Therefore, these protein translational modifications were investigated in this study to be considered for possible manufacture in the future.

The predicted low solubility of the vaccine construct (0.291) represents a practical consideration for downstream formulation and production, as solubility can influence protein stability, formulation performance, and manufacturing processes. This aspect may be effectively addressed through liposomal or lipid nanoparticle–based formulation strategies, which have demonstrated strong potential for improving the stability, delivery, and bioavailability of antigens with limited solubility. Encapsulation in liposomal carriers can protect the antigen from aggregation, enhance cellular uptake, and support efficient intracellular antigen expression, thereby reducing the functional impact of solubility constraints on in vivo performance. Although experimental validation remains necessary, liposomal delivery offers a promising and scalable solution to support the translational feasibility of the vaccine platform.

One limitation of the current study was the system's processing power; a more robust graphics processing unit (GPU) would have enabled the analysis of a greater volume of data. Additionally, the selection of a linker to stabilize the 3D structure of our designed vaccine was another constraint, suggesting a need for further research into linkers. Further mRNA technology faces limitations, including antigen variability and genetic differences that affect vaccine efficacy. Additional challenges include post‐translational modifications, population‐specific responses, and complex, costly manufacturing. Although the immune simulations do not explicitly model exhaustion, Treg induction, PD‐1 dynamics, or cytokine storm risk, they provide valuable preliminary insights into the vaccine's immunogenic potential. These results guide experimental design and highlight key targets for TNBC immunotherapy, serving as a strong foundation for future in vitro and in vivo validation.

Future validation efforts will follow a structured experimental pipeline to confirm the biological relevance of LRP6 and evaluate vaccine performance. This roadmap includes validating LRP6 expression in TNBC cell lines at both transcript and protein levels, assessing dendritic cell uptake and antigen presentation in vitro, and measuring antigen‐specific cellular immune responses using IFN‐γ ELISPOT assays. Vaccine efficacy will then be tested in a syngeneic TNBC mouse model, with tumor progression and survival outcomes monitored to determine therapeutic impact. In parallel, a lipid nanoparticle–based delivery strategy will be developed and optimized to enhance antigen stability, cellular delivery efficiency, and immune activation. Together, these steps provide a comprehensive framework for experimental validation and translational advancement.

## 5 Conclusion

Triple‐negative breast cancer remains a major clinical challenge due to its aggressive behavior and lack of targeted therapies. In this study, we designed and computationally validated an innovative mRNA vaccine targeting LRP6, a key receptor implicated in TNBC progression. The vaccine demonstrated high predicted antigenicity, non‐allergenicity, structural stability, and broad global population coverage. Computational analyses, including immune simulations and molecular docking, indicated its capacity to stimulate durable adaptive and innate immune responses. Despite limitations, such as computational resource constraints, further research is needed to validate these findings in preclinical and clinical settings. Nonetheless, the results of this study provide a strong foundation for developing an effective LRP6‐targeted mRNA vaccine for TNBC.

## Author Contributions

**Mohammadreza Heidari:** conceptualization, methodology, supervision, writing – review and editing. **Pooriya Teimoori:** conceptualization, methodology, investigation, writing – original draft.

## Funding

The authors have nothing to report.

## Disclosure

The authors declare that no AI‐generated images or AI‐generated data were used in this study. AI‐assisted tools were used solely for language editing and grammar refinement during manuscript preparation. These tools did not generate scientific content, influence study design, data analysis, interpretation of results, or conclusions. All analyses, interpretations, and final decisions were made by the authors.

## Ethics Statement

All authors listed have made significant contributions to the conception, design, execution, and interpretation of the study. No contributors have been excluded from authorship. All data are accurate and were collected and analyzed in accordance with accepted scientific standards. Raw data and supporting materials are available upon request.

## Conflicts of Interest

The authors declare no conflicts of interest.

## Supporting information


**Table S1:** 3D modeling validation result of constructed vaccine.

## Data Availability

The data that support the findings of this study are available from the corresponding author upon reasonable request.
