# Supplementary material for: Computational Design of an mRNA Vaccine Targeting LRP6 for Triple‐Negative Breast Cancer Therapy
Source: Cancer Rep (Hoboken). 2026 May 5;9(5):e70567. doi: 10.1002/cnr2.70567 (PMC13143567; doi:10.1002/cnr2.70567)
Supplement: Supplementary file 1 — Table S1: 3D modeling validation result of constructed vaccine. [file CNR2-9-e70567-s001.docx]

| **ProCheck** | **Ramachandran Plot** | Residues in most favored regions | 312 | 91.0 % |
| --- | --- | --- | --- | --- |
|  |  | Residues in additional allowed regions | 25 | 7.3% |
|  |  | Residues in other regions | 6 | 1.8 % |
|  | **ERRAT** | Overall quality factor | 90.553 | |
|  | **Verify 3D** | | 78.17% | |
| **Antigenicity** | | | 0.6596 | |
| **Allergenicity** | | | PROBABLE  NON-ALLERGEN | |
| **Toxicity** | | | Non-Toxin | |

Supplementary Table 1. 3D modeling Validation result of constructed vaccine.
